# Regional Variations in the Prevalence of Risk Factors and Non-Communicable Diseases in Papua New Guinea: A Scoping Review

**DOI:** 10.3390/ijerph22010102

**Published:** 2025-01-14

**Authors:** Bobby Porykali, Ryley Gronau, Phyllis Tran, Juliana Chen, Margaret Allman-Farinelli, Anna Rangan, Shelina Porykali, Robin Oge, Hans Nogua, Alyse Davies

**Affiliations:** 1 Aboriginal and Torres Strait Islander Health Program, George Institute for Global Health, Sydney, NSW 2000, Australia; 2 Faculty of Medicine, School of Population Health, University of New South Wales, Sydney, NSW 2052, Australia; 3 Discipline of Nutrition and Dietetics, Susan Wakil School of Nursing and Midwifery, Faculty of Medicine and Health, The University of Sydney, Sydney, NSW 2006, Australiaalyse.davies@sydney.edu.au (A.D.); 4 Charles Perkins Centre, The University of Sydney, Sydney, NSW 2006, Australia; 5 School of Health Sciences and Social Work, Griffith University, Gold Coast, QLD 4222, Australia; 6 National Capital District Provincial Health Authority, Port Moresby 121, Papua New Guinea; 7 Port Moresby General Hospital, National Capital District, Port Moresby 121, Papua New Guinea

**Keywords:** Papua New Guinea, Western Pacific region, Healthy Islands, non-communicable diseases, NCDs, modifiable behavioural risk factors

## Abstract

Often referred to as ‘the last unknown’, Papua New Guinea’s largely unexplored environments across its four distinct regions, the Highlands, New Guinea Islands, Momase, and Southern, exhibit remarkable diversity. Understanding this diversity is significant in contextualising the risk factors associated with developing non-communicable diseases. This review aims to map and summarise the literature to provide region-specific prevalence data for risk factors and non-communicable diseases. Four databases and grey literature were searched. Two reviewers completed the screening and data extraction. Twenty-one studies were included, with five reporting the data by region and the remaining reporting the data nationwide. Six studies reported on risk factors, thirteen reported on non-communicable diseases, and two reported on risk factors and non-communicable diseases. The Southern region, which includes the Capital, Port Moresby, reported the highest prevalence for most risk factors: anthropometric (overweight, obesity, and waist circumference), lifestyle (betel nut, alcohol, unhealthy diet, and stress), and biochemical (cholesterol, triglycerides, HbA1c, and metabolic syndrome). The findings of this review highlight the limited evidence base for region-specific risk factor data and the lack of objective diagnosis of non-communicable diseases. There were variations in the prevalence of specific risk factors by region; however, the Southern region stands out as requiring immediate attention for health promotion program interventions.

## 1. Introduction

**Figure 1:**
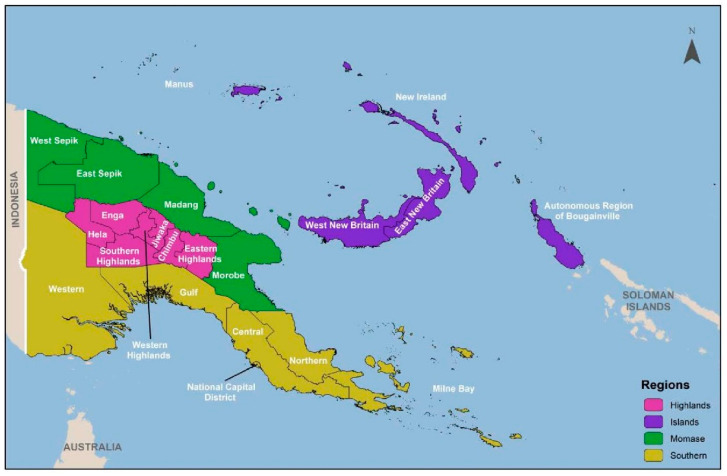
Map of Papua New Guinea. Source: Papua New Guinea Demographic and Health Survey 2016–18 [14].

Papua New Guinea (PNG) exhibits remarkable diversity, characterised by abundant resources, varied natural landscapes, and a multitude of cultures, with approximately 800 documented living languages [1]. The country presents an enigmatic complexity that eludes uniform depiction, with certain regions remaining uncharted, even with modern mapping and aerial navigation tools, which is why the country is often referred to as the ‘the last unknown’. The internal terrain is mostly mountainous, with coastal lowlands and islands scattered on the coast. There are four regions and 22 provinces. Port Moresby, located in the Southern region, was made the capital city when PNG gained independence in 1975 [2]. The mountainous Highlands region occupies the central part of PNG, the New Guinea Islands is located northeast of the mainland, and the Momase region, located in the northwest, includes urban areas of Lae and Madang (Figure 1).

The nutrition transition towards Western diets and lifestyles has been documented in PNG [3], although over 80% of the population still live in remote villages and largely maintain a subsistence way of life [4]. Those living in urban areas have direct exposure to the nutrition transition, which is reflected in the composition of their diet being higher in energy, protein, and fat compared to their rural counterparts [1]. Data from food imports show that the demand for international processed foods and sugar-sweetened beverages (SSBs) has increased over time [5], and processed food sales are high [6]. SSBs are commonly consumed throughout all regions in PNG and even firmly extending into traditional ceremonies, cultural gatherings, and events. The shifting dietary habits of the population are also being influenced by cash cropping, where traditional garden foods, coffee, cocoa, and betel nut are generating income for the purchase of store-bought foods [3].

As observed in other World Health Organisation (WHO) Western Pacific Regions, nutrition transitions have been associated with a rise in non-communicable disease risk factors and non-communicable diseases (NCDs) [7]. PNG is part of the WHO Healthy Islands Initiative, which has a focus on addressing NCDs [8]. NCDs include type 2 diabetes mellitus (T2DM), cardiovascular disease (CVD), chronic respiratory diseases, and cancers [9]. The lifestyle risk factors associated with NCDs include an unhealthy diet, physical inactivity, smoking, and the harmful use of alcohol [9]. The frequent social chewing of betel nut quid in PNG, beyond that of what was reserved for customary usage, has been associated with oral cancer [10]. In 2018–20, NCD-related deaths in PNG accounted for 47% of total deaths, showing a 10% increase over the past 50 years [11]. Since PNG has the largest population amongst the Pacific Island nations, exceeding 10 million [12], this places a significant proportion of people within the region at risk.

A previous review reported on the prevalence of NCDs in adults (over 15 years) and their associated risk factors and reported on studies published prior to the year 2016 [13]. Given the diversity of PNG, an understanding of risk factors and NCDs by region is needed to provide direction for future and targeted lifestyle disease programs. This review aims to map and summarise the literature to provide region-specific prevalence data for risk factors and non-communicable diseases and provide an update on the nationwide prevalence data.

To contextualise this research in the context of non-communicable diseases and health systems in PNG, a positionality statement is important. Four members of the research team (R.O., H.N., B.P., S.P.) have heritage from PNG. R.O. and H.N. reside in PNG and are specialist medical doctors. R.O. is acting CEO at the Provincial Health authority in the National Capital District. B.P. is a senior research fellow with expertise in population health in PNG, while S.P. is a new graduate dietitian and PhD candidate. A.D. has family ties to PNG and is a lecturer in the discipline of nutrition and dietetics.

## 2. Materials and Methods

### 2.1. Protocol and Registration

The protocol for this scoping review was developed and registered on the Open Science Platform (https://osf.io/pvkg8/ (accessed on 22 May 2023)). The findings are reported in accordance with PRISMA extension for scoping reviews and Joanna Briggs Institute updated methodological guidance for scoping reviews [15,16].

### 2.2. Inclusion Criteria

#### 2.2.1. Participants

Studies including Pacific Islander adults (>15 years) by region were considered as well as nationwide studies. If Papua New Guineans constituted less than 50% of the sample population or if the data could not be distinguished from other populations, the study was omitted.

#### 2.2.2. Concept

Studies that considered the prevalence of risk factors and NCDs were included. The risk factors were grouped into four categories: (1) anthropometric (underweight, overweight, obesity, waist circumference (WC), waist-to-hip ratio (WHR)); (2) lifestyle (smoking, betel nut chewing, alcohol, insufficient physical activity, unhealthy diet, stress); (3) biochemical and/or metabolic syndrome (METs) (micronutrient deficiencies, total cholesterol (TC), low-density lipoprotein cholesterol (LDL), high-density lipoprotein cholesterol (HDL), triglycerides (TG), blood glucose, HbA1c, METs; (4) physiological (prehypertension, hypertension). The NCDs were grouped into four categories: T2DM, CVD, chronic respiratory disease, and cancer. Cancers were included if diet was associated with a probable or convincing increased risk in accordance with the World Cancer Research Fund (WCRF) [17].

#### 2.2.3. Context

This review included papers that reported by region, Southern region (SR), Highlands region (HR), Momase region (MR), and New Guinea Islands (NGI), and nationwide.

#### 2.2.4. Types of Evidence Sources

Population-based studies published from 2016 were included in order to provide an update from a previous review on the prevalence of NCDs and their risk factors of adults in PNG [13]. All primary study designs, government reports, websites, and studies that contained secondary analysis of primary data were included. Conference abstracts, thesis, systematic reviews, reviews with no secondary analysis of primary data, meta-analyses, policy papers, and expert opinions were excluded. Studies were limited to human studies, and language was restricted to English.

### 2.3. Information Sources and Search

The review team drafted the search strategy in consultation with an experienced university librarian. Relevant studies were identified using the following four electronic databases: Medline, Embase, Global Health, and Scopus. Grey literature searches were conducted by using the advanced search function on Google Scholar (limited to the first 200 records). A citation search of articles included for full-text screening was performed to scan for additional documents. See Supplementary Table S1 for the final MEDLINE search strategy conducted on 18 March 2023. The search strategy for each database, including the number of records, can be found in the published protocol.

### 2.4. Selection Process

All documents retrieved from the search were exported to EndNote20 citation management software to remove duplicates. The citations were imported into Covidence to be independently screened by two reviewers (R.G. and P.T.) in accordance with the eligibility criteria. The study selection process included title and abstract screening, followed by full texts. For both stages, any discrepancies between the two reviewers were resolved by a third reviewer (A.D., J.C. and B.P.).

### 2.5. Data Charting Process and Data Items

Data extraction was conducted independently by two reviewers (R.G. and P.T.) using a standardised data charting form for scoping reviews [16]. The following data were extracted: first author and year, dataset, region, population characteristics, sample size, year conducted, prevalence and/or incidence for risk factors (anthropometric, lifestyle, biochemical and/or METs, and physiological), and NCDs (T2DM, CVD, chronic respiratory disease, and cancer).

### 2.6. Synthesis of Results

The results are displayed in a tabulated format, supplemented by a narrative summary to describe the results in relation to the aims of the scoping review.

## 3. Results

### 3.1. Search Results

**Figure 2:**
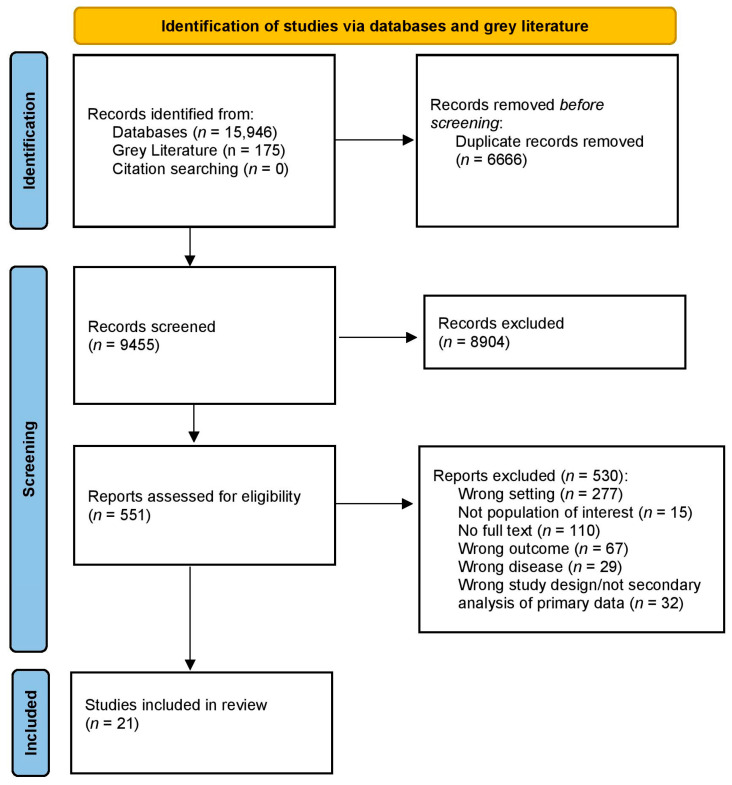
PRISMA flow diagram of record identification and study selection for a scoping review on the prevalence of non-communicable diseases and associated risk factors in Papua New Guinea.

Databases, grey literature, and citation searching identified a total of 16,121 records. A total of 6666 duplicates were removed. The titles and abstracts of 9455 were screened, and 8904 records were excluded. A full-text review of 551 records was carried out to assess eligibility, and 530 were excluded. A total of 21 studies were included in this scoping review (Figure 2).

### 3.2. Study Characteristics

The study characteristics and the prevalence of risk factors and NCDs in PNG are presented in Table 1. The 21 included studies were published between 2017 and 2023 [18,19,20,21,22,23,24,25,26,27,28,29,30,31,32,33,34,35,36,37,38] but included data between 2005 and 2019. The population size of the studies varied between 79 and 21,970 individuals [18,19,20,21,23,24,28], 16,021 households [22], with 13 not reporting population size [25,26,27,29,30,31,32,33,34,35,36,37,38]. While two studies used self-collected data [18,19], nineteen used population datasets, including the PNG Integrated Health and Demography Surveillance System [20,21], PNG Demographic and Health Survey [22,23], Global Youth Tobacco Survey [24], Global Burden of Disease [25,26], WHO Global Health Observatory [26,27], Biomarkers Reflecting Inflammation and Nutritional Determinants of Anemia project [28], and GLOBOCAN [29,30,31,32,33,34,35,36,37,38]. The majority of the studies reported nationwide data [23,24,25,26,27,28,29,30,31,32,33,34,35,36,37,38], while two studies reported by region [18,19] or multiple regions [20,21,22]. No studies reported by urban and rural areas. A total of six studies reported on risk factors [18,21,22,23,24,28], thirteen reported on NCD [19,25,27,29,30,31,32,33,34,35,36,37,38], and two reported risk factors and NCDs [20,26].

### 3.3. Prevalence of Risk Factors

#### 3.3.1. Anthropometric

A total of four studies reported on anthropometric data (measured data *n* = 3) [18,20,21], one study reported on underweight [18], one reported on overweight [20], two reported on obesity [20,26], one reported on WC [21], and two reported on WHR [20,21].

For underweight (<18.5 kg/m^2^), one study, using data from 2015 [18], reported a prevalence of 23% in the SR.

For overweight (≥25–29.9 kg/m^2^), one study reported on three regions (SR, HR, MR), using data between 2013 and 2014 [20], with an overall prevalence of 19%, similar between genders. The highest prevalence was in the SR (25%).

For obesity (BMI ≥ 30 kg/m^2^), one study in three regions (SR, HR, MR), using data between 2013 and 2014 [20], reported an overall prevalence of 11%, higher for females (15%) than males (6%) and highest in the SR (22%). One nationwide study, using data from 2016 [26], reported a higher prevalence for females (26%) than males (17%).

For WC (≥94 cm male, ≥80 cm female), one study reported on three regions (SR, HR, MR), using data between 2013 and 2014 [21], with the overall prevalence higher for females (52%) than males (9%) and highest in the SR (44%).

For WHR (≥0.90 males, ≥0.85 females), two studies reported on three regions (SR, HR, MR), using data between 2013 and 2014 [20,21]. The overall prevalence was 68%, with females higher (72–73%) than males (61–62%) and highest in the HR (89–90%).

#### 3.3.2. Lifestyle

A total of six studies reported on lifestyle data [20,21,22,23,24,26]. Five studies reported on smoking [20,21,22,23,24], three reported on betel nut use [20,21,23], two reported on alcohol consumption [20,21], two reported on insufficient physical activity [20,26], two reported on diet [20,21], and one reported on stress [20].

Smoking was defined as daily use of any form of tobacco product [20,21], current cigarette smoking [22], current smokeless tobacco [23], or cigarette smoking (past 30 days) [24]. Two studies reported on three regions (SR, HR, and MR), using data between 2013 and 2014 [20,21], with an overall prevalence of 48%, higher for males (between 65 and 69%) than females (between 22 and 29%). Three studies reported on smoking in the SR [20,21,22]. The prevalence was between 36 and 41%, using data between 2013 and 2014 [20,21], and 21% for breastfeeding adults, using data between 2016 and 2018 [22]. There was a higher prevalence for males (66%) than females (20%), using data between 2013 and 2014 [20]. Three studies reported on smoking in the HR [20,21,22]. The prevalence was between 40 and 50%, using data between 2013 and 2014 [20,21], and 26% for breastfeeding adults, using data between 2016 and 2018 [22]. There was a higher prevalence for males (69%) than females (28%), using data between 2013 and 2014 [20]. Three studies reported on smoking in the MR [20,21,22]. The prevalence was between 50 and 52%, using data between 2013 and 2014 [20,21], and 33% for breastfeeding adults, using data between 2016 and 2018 [22]. There was a higher prevalence for males (71%) than females (37%), using data between 2013 and 2014 [20]. One study reported on the NGI, with a 20% prevalence for breastfeeding adults, using data between 2016 and 2018 [22]. Three nationwide studies, using data between 2016 and 2018, reported a prevalence of 65% [23], 22% (breastfeeding adults) [22], and 25% (under the age of 18 years) [24].

Betel nut chewing was defined as any amount of betel nut chewing within the last 30 days [20,21] or daily betel nut (with or without tobacco) [23]. Two studies reported on three regions (SR, HR, MR), using data between 2013 and 2014 [20,21], with a 74% prevalence and similar between genders. The highest prevalence was in the SR (93–94%). One nationwide study, using data between 2016 and 2018, reported an overall prevalence of 65% [23].

Alcohol consumption was defined as alcohol consumption (any amount) within the last 30 days [20,21]. Two studies reported on three regions (SR, HR, MR), using data between 2013 and 2014 [20,21], with an overall prevalence of 23%, higher for males (39–42%) than females (8%). The highest prevalence was in the SR (43–45%). Males had a higher prevalence than females across all regions.

Insufficient physical activity was defined as <75 or 150 min per week on vigorous and moderate physical activity [20,26], with one study developing physical activity questions for the study [20] and the other not reporting the measurement tool [26]. One study reported on three regions (SR, HR, and MR), using data between 2013 and 2014 [20], with an overall prevalence of 20%, higher for females (24%) than males (16%). The highest insufficient physical activity prevalence was in the MR (34%). One nationwide study, using data from 2016 [26], reported a higher prevalence for females (18%) than males (11%).

Two studies reported on a typical week’s food consumption, overall and by region (SR, HR, and MR), using data between 2013 and 2014 [20,21]. For vegetables, 35% did not consume root and 42% did not consume green vegetables at least five days in a typical week, with the lowest intakes in the SR [20]. Fruit was not consumed by 87% at least five days in a typical week, with the lowest intakes in the MR [20]. A total of 23% of males and 25% of females consumed fruit and vegetables less than 5 days per week, with the highest in the SR (59%) [21]. Fresh protein was not consumed by 72% at least five days in a typical week, with the highest in the HR. Canned protein was consumed at least 5 days in a typical week by 23%, with the highest in the SR [20]. Sugar (>6 teaspoons daily) and sugary drink (>3 days per week) consumption was 17% and 6%, respectively [20], and a high sugar intake (both sugar and sugary drinks) was highest for males [21] and the SR [20,21]. Fried food (home or purchased) was consumed by 67% of males and 66% of females and was higher in both the HR (97%) and SR (92%) [21]. Both males (70%) and females (68%) added salt or Maggie stock cubes on food, with the highest in both the HR and SR [21].

For self-reported stress (currently feeling stressed), one study reported on three regions (SR, HR, and MR), using data between 2013 and 2014 [20], with an overall prevalence of 32%, similar between genders. The highest prevalence was in the SR (46%). The prevalence was similar between males and females across all regions.

#### 3.3.3. Biochemical and/or Metabolic Syndrome (METs)

A total of five studies reported on biochemical and/or METs [18,20,21,26,28]. Two studies reported on micronutrient deficiencies [18,28], three reported on TC [20,21,26], two reported on HDL [20,21], one reported on TG [21], two reported on HbA1c [20,21], and one reported on METs [21]. No studies reported on LDL or blood glucose.

For micronutrient deficiencies, one study reported on the SR, using data from 2015, with an anaemia prevalence of 35% and iodine deficiency of 80% [18]. One nationwide study, using data from 2005 [28], reported an anaemia prevalence of 36%, iodine deficiency of 8%, and vitamin A deficiency of 1%.

For elevated TC, all three studies used a cut off >6.2 mmol/L. Two studies reported on three regions (SR, HR, MR), using data between 2013 and 2014 [20,21], with an overall prevalence of 17% and the highest in the SR (23–24%). One nationwide study, using data from 2008, reported a similar prevalence between genders (8–10%) [26].

For low HDL, both studies used a cut off <1 mmol/L for males and <1.3 mmol/L for females. Two studies reported on three regions (SR, HR, MR), using data from 2013 to 2014 [20,21], with an overall prevalence of 55% and a higher prevalence for females (64–65%) than males (42–44%). The highest prevalence was reported in the MR (62–63%).

For elevated TG, one study reported on three regions (SR, HR, MR), using data between 2013 and 2014 and a cut off ≥2.3 mmol/L [21]. A similar overall prevalence was reported between genders (54–56%), and the highest prevalence was in the SR (62%).

For HbA1c, using the cut off ≥5.7–6.4% (pre-diabetes), one study reported on three regions (SR, HR, MR), using data between 2013 and 2014 [21]. The overall prevalence was 15%, similar between genders (13–17%). The highest prevalence was in the SR (26%), with females higher (31%) than males (19%). For HbA1c, using the cut off (≥6.5%, T2DM), two studies reported on three regions (SR, HR, MR), using data between 2013 and 2014 [20,21]. The overall prevalence was 3%, similar between genders, with the highest prevalence in the SR (9%).

For METs, one study reported on three regions (SR, HR, MR), using data between 2013 and 2014 [21], with females (30%) higher than males (6%) and the highest prevalence in the SR (27%).

#### 3.3.4. Physiological

A total of three studies reported on physiological data [20,21,26]. One study reported on prehypertension [20], and three reported on hypertension [20,21,26].

For prehypertension (cut off SBP > 120–139.9 mmHg and/or DBP 80–89 mmHg), one study reported on three regions (SR, HR, MR), using data between 2013 and 2014 [20]. The overall prevalence was 46%, higher for males (55%) then females (38%). The prevalence was highest in the HR (55%).

For hypertension, two studies on three regions (SR, HR, MR), using data between 2013 and 2014 (using different cut offs) [20,21], reported an overall prevalence of 16%, higher for males (19–38%) than females (13–28%). The highest prevalence was in the SR (22–40%) and HR (22–41%). One nationwide study (cut off ≥140/90 mmHg), using data from 2015 [26], reported a similar prevalence between males (25%) and females (26%).

### 3.4. Prevalence or Incidence of NCDs

#### 3.4.1. T2DM

A total of four studies reported on T2DM [19,20,26,27]. One study on three regions (SR, HR, MR), using data between 2013 and 2014 [20], reported a 1% overall prevalence (self-report), and another from the SR (National Capital District), using data from 2017 [19], reported an 8% prevalence (known and newly diagnosed). Two nationwide studies, using data from 2014 [26,27], reported a 15% prevalence, similar between genders.

#### 3.4.2. CVD

A total of two studies reported on CVD in adults [20,26]. One study on three regions (SR, HR, MR), using data between 2013 and 2014 [20], reported an overall prevalence of 0.4% for stroke and 1.0% for heart disease (self-report). One nationwide study, using data from 2010, reported on stroke prevalence/100,000 (391/100,000) and incidence/100,000 person-years (159/100,000) [26].

#### 3.4.3. Chronic Respiratory Disease

One study on three regions (SR, HR, MR), using data between 2013 and 2014 [20], reported an overall prevalence of 2%, with the highest in the SR (4%).

#### 3.4.4. Cancer

A total of 12 studies reported on cancer prevalence [20,25,29,30,31,32,33,34,35,36,37,38]. One study on three regions (SR, HR, MR) reported a 0.3% cancer prevalence (self-report), using data between 2013 and 2014 [20]. Three studies reported on the 2012 GLOBOCAN data for incidence (age standardised rate) for liver (11/100,000) [29], lip (9/100,000) [30], oral cavity (11/100,000) [30], parotid and salivary glands (3/100,000) [30], and kidney cancer (1/100,000) [31]. Six studies reported on the 2018 GLOBOCAN data for incidence (age standardised rate) for liver (12/100,000) [36], oral cavity (30/1,000,000 for male and 21/100,000 for female) [32], pancreas (2/100,000) [33], lip and oral cavity (20/100,000) [34,35], and gastric cancer (9/100,000) [38]. Another study reported on the Global Burden of Disease data and reported a 1.0% prevalence and 2.0% incidence for liver cancer (2019) [25].

## 4. Discussion

This scoping review provides region differences on the prevalence of risk factors and NCDs in PNG for studies published between 2016 and 2023. For anthropometric risk factors, the SR had the highest prevalence for overweight, obesity, and WC. In terms of lifestyle risk factors, smoking and insufficient physical activity were highest in the MR, and betel nut chewing, alcohol consumption, and stress were highest in the SR. The SR and HR reported a high prevalence for the consumption of sugar/sugary drinks, salt, and fried foods. The consumption of vegetables (root and green) was lowest in the SR, and fruit consumption was lowest in the MR. For biochemical risk factors, the SR reported the highest prevalence for TC, TG, HbA1c, and METs. For physiological risk factors, prehypertension and hypertension were highest in the HR. The prevalence of NCDs (T2DM, CVD, and chronic respiratory disease) was highest in the SR but relied on self-reported diagnosis. The highest incidence of cancer was for the liver, lip, and oral cavity.

While the trend for deaths related to NCDs is increasing [39], this is not being captured in the prevalence data as many people living with NCDs in PNG remain undiagnosed (around 97%) and are not known to the health services [40,41]. This may be attributed to a combination of factors, including low-level health literacy, limited accessibility and affordability of services, and an under-resourced healthcare system. Compared to the prevalence of NCDs from the review published in 2020 [13], the rates seem to show a decline, but it is important to note that the studies included in our review were based on self-reported diagnosis, and therefore, the findings need to be interpreted with caution.

While PNG is making progress in improving its health data management systems, structural challenges remain, particularly around infrastructure, workforce development, and data management practices. In this context, from clinical experiences, a major challenge is that the electronic national health information system (eNHIS) in PNG captures minimal risk factor and prevalence data. Changes to this information system to update data capture can take up to five years. Provincial Health Authorities (PHA) are mandated bodies that provide healthcare across each of the respective 22 provinces. PHA need integrated robust health information systems; however, at a clinical level, risk factor data including NCD data are manually captured through registry books. This is inadequate for programs to conduct NCD and its risk factor surveillance and manage patients with chronic NCDs. It is crucial that PHA lifestyle disease programs receive appropriate investment of resources and support. In contextualising information by regions, it is important to understand that the National Capital District (includes Port Moresby, the capital city) is within the SR and the area experiencing the most rapid urbanisation [1]. Other areas within the SR, such as the Gulf, Western, Central, and Miline Bay provinces, have a predominantly rural population who survive mostly on subsistence farming. Similarly, the city of Lae within the MR is another area experiencing rapid urbanisation and constitutes the largest PHA by geographic area of service delivery.

PNG is experiencing a double burden of malnutrition, which is defined by the coexistence of undernutrition, over nutrition, and diet-related NCDs [42,43]. For adults, overweight, obesity, and visceral adiposity were most prevalent in the SR, likely due to a combination of factors, including a higher socioeconomic status due to greater employment opportunities and the increased access to imported, high-energy foods and beverages. Similar to the previous review [13], smoking showed a high prevalence of up to 65%, higher in males and the MR. While there is significant health risks, such as the development of oral cancers associated with betel nut chewing with lime and mustard stick [44], it remains prevalent, highest in the SR, given its cultural significance, narcotic and stimulant effect, and ease of accessibility due to the increase in sales within the informal sector. With the cost associated with the consumption of alcohol, it has been suggested that intakes are higher in urban areas and those that engage in formal employment [13], which is in line with our findings of being highest in the SR and among males. Stress was only identified in one study and highest in the SR and was suggested to be related to the dependence on the cash-based economy for acquiring goods and services [20].

The significance of a healthy diet and physical activity cannot be understated in the prevention and management of NCDs. Only two studies [20,21] in this review reported on diet; the SR and HR had the highest prevalence for the consumption of sugar/sugary drinks, salt (addition of Maggie stock cubes), and fried foods, and the SR had the lowest consumption of vegetables, which can increase the risk of developing NCDs. Only two studies reported on physical inactivity; the highest prevalence was among women, similar to other Pacific Island nations [45]. Addressing the cultural, economic, and behavioural determinants of NCDs is essential for effective prevention and management strategies in PNG [46]. With only two health and nutrition promotion programs targeting risk factors and NCDs in MR and NGI of PNG [46], prioritising cultural integrated interventions that promote and educate on a healthy diet, portion sizes, and the importance of physical activity is crucial for mitigating the burden of NCDs in the population and especially for those living in the SR, where risk factors are more prevalent.

Collecting and reporting on biochemical and physiological prevalence data are essential for gaining a comprehensive understanding of the health status and disease burden within a population, but these were also not widely reported. Findings from our review showed that the SR had the highest prevalence for a range of biochemical measures (TC, TG, HbA1c) and METs, whilst the HR had the highest prevalence for prehypertension and hypertension. This ties in with the region-specific diet risk factor data in which the SR and HR had the highest intakes of sugar/sugary foods, salt, and fried foods, which are foods that are well known to have an impact on these biochemical markers. Similar findings were also shown in a recent study in Fiji, where salt and sugar are commonly consumed [47]. Having a comprehensive understanding of the biochemical and physiological data informs the development of targeted public health strategies and healthcare policies tailored to the specific needs of the population, ultimately leading to improved health outcomes and reduced morbidity and mortality from NCDs.

A strength of this review was that data were reported by region and gender, where possible, to understand the distribution of risk factors and NCDs. Four authors on this publication have heritage from PNG and are able to contextualise this research in the context of PNG, two of whom have extensive in-country clinical experience. The limitations include the reliance on cross-sectional studies. The majority of the studies reported nationwide data rather than by region. There were limited studies (only eight out of the 21 included studies) that reported on select risk factors, and the variations in reporting each risk factor made it difficult to compare results between studies. The data relied on self-reported diagnosis for the prevalence of NCDs, and therefore, we were not able to provide an update on nationwide trends over time with confidence. Even though we included studies that were published since 2016, several studies included data from 2005 and, therefore, may not represent the most recent developments or changes since the previous review published in 2020. We acknowledge that, while there are over 800 living languages in PNG, we restricted studies to manuscripts written in English, given it is the official language of education and government in PNG.

## 5. Conclusions

The findings of this review highlight the limited evidence base for region-specific risk factor data and the lack of objective diagnosis of NCDs. Further, it underscores the critical necessity for enhancing health management data systems and routine screening to accurately capture the prevalence of associated risk factors and NCDs across regions in PNG. There were variations in the prevalence of specific risk factors by region; however, the SR stands out as requiring immediate attention for health promotion program interventions.

## Figures and Tables

**Table 1:** Characteristics of included studies and the prevalence of risk factors and non-communicable diseases in Papua New Guinea.

Study Characteristics							Prevalence ‡
First Author, Year, Ref	Dataset	Region	Population, Age (Years), Sample Size (n)	Year Conducted	Outcome	Type	Total (T), Male (M), Female (F)
Goris, 2017 [18]	Self-collected data from a prospective cross-sectional study	Southern (Gulf Province, Kerema District), 10 villages in Kotidanga Local Level Government	*n* = 79 (non-pregnant women)	2015	Risk Factor (anthropometric)	Underweight BMI (<18.5 kg/m^2^)	Southern: 23% (T)
					Risk Factor (biochemical and/or metabolic syndrome)	Anaemia (Hb < 120 g/L)	Southern: 35% (T)
						Iodine deficiency	Southern: 80% (T)
Burnett, 2019 [19]	Self-collected data from a cross-sectional population-based survey	Southern (National Capital District)	Age: ≥50 *n* = 1186	2017	NCD	Diabetes (known and newly diagnosed, newly diagnosed if not previously diagnosed with diabetes but had a random BG ≥ 11.1 mmol/L)	Southern: 8% (T), 8% (M), 8% (F)
Rarau, 2017 [20]	PNG Integrated Health and Demography Surveillance System	Southern (Central Province—West Hiri), Highlands (Eastern Highlands Province—Asaro), Momase (Madang Province—Karkar Island)	Age: (15–65 years), *n* = 772 (overall) *n* = 266 (West Hiri), *n* = 254 (Asaro), *n* = 252 (Karkar Island)	2013–2014	Risk Factor (anthropometric)	Overweight (BMI ≥ 25–29.9 kg/m^2^)	Overall: 19% (T), 19% (M), 19% (F) Southern: 25% (T), 29% (M), 22% (F) Highlands: 23% (T), 23% (M), 24% (F) Momase: 8% (T), 5% (M), 10% (F)
						Obesity (BMI ≥ 30 kg/m^2^)	Overall: 11% (T), 6% (M), 15% (F) Southern: 22% (T), 13% (M), 29% (F) Highlands: 6% (T), 2% (M), 10% (F) Momase: 3% (T), 2% (M), 4% (F)
						WHR (≥0.9 cm (M)/≥0.85 cm (F))	Overall: 68% (T), 62% (M), 73% (F) Southern: 52% (T), 45% (M), 58% (F) Highlands: 90% (T), 98% (M), 82% (F) Momase: 62% (T), 39% (M), 81% (F)
					Risk Factor (lifestyle) (self-reported)	Smoking (daily)	Overall: 48% (T), 69% (M), 29% (F) Southern: 41% (T), 66% (M), 20% (F) Highlands: 50% (T), 69% (M), 28% (F) Momase: 52% (T), 71% (M), 37% (F)
						Betel nut (last 30 days)	Overall: 74% (T), 73% (M), 75% (F) Southern: 94% (T), 90% (M), 96% (F) Highlands: 55% (T), 58% (M), 52% (F) Momase: 75% (T), 74% (M), 76% (F)
						Alcohol (self-reported) (last 30 days)	Overall: 23% (T), 39% (M), 8% (F) Southern: 43% (T), 69% (M), 21% (F) Highlands: 22% (T), 37% (M), 6% (F) Momase: 7% (T), 16% (M), 0% (F)
						Insufficient physical activity (<75 or 150 min/week on vigorous and moderate)	Overall: 20% (T), 16% (M), 24% (F) Southern: 23% (T), 18% (M), 27% (F) Highlands: 6% (T), 5% (M), 8% (F) Momase: 34% (T), 29% (M), 37% (F)
						Food consumption Root vegetables (did not consume for at least 5 days in a typical week)	Overall: 35% (T) Southern: 83% (T) Highlands: 17% (T) Momase: 2% (T)
						Greens (did not consume for at least 5 days in a typical week)	Overall: 42% (T) Southern: 86% (T) Highlands: 34% (T) Momase: 2% (T)
						Fruit (did not consume for at least 5 days in a typical week)	Overall: 87% (T) Southern: 76% (T) Highlands: 87% (T) Momase: 100% (T)
						Fresh protein (did not consume for at least 5 days in a typical week)	Overall: 72% (T) Southern: 52% (T) Highlands: 98% (T) Momase: 67% (T)
						Canned protein (consumed for at least 5 days in a typical week)	Overall: 23% (T) Southern: 52% (T) Highlands: 10% (T) Momase: 7% (T)
						Sugar (>6 teaspoons daily)	Overall: 17% (T) Southern: 20% (T) Highlands: 20% (T) Momase: 11% (T)
						Sugary drinks (>3 days/week in a typical week)	Overall: 6% (T) Southern: 13% (T) Highlands: 4% (T) Momase: 0% (T)
						Purchased fried food (consumed for at least 5 days in a typical week)	Overall: 2% (T) Southern: 2% (T) Highlands: 6% (T) Momase: 0% (T)
						Home fried food (consumed for at least 5 days in a typical week)	Overall: 20% (T) Southern: 16% (T) Highlands: 43% (T) Momase: 0% (T)
						Stock cube (consumption for 7 days/week)	Overall: 1% (T) Southern: 1% (T) Highlands: 0% (T) Momase: 1% (T)
						Salt directly on food (consumption for 7 days/week)	Overall: 47% (T) Southern: 61% (T) Highlands: 57% (T) Momase: 22% (T)
						Stress (currently feeling stressed)	Overall: 32% (T), 33% (M), 31% (F) Southern: 46% (T), 43% (M), 48% (F) Highlands: 44% (T), 47% (M), 41% (F) Momase: 5% (T), 4% (M), 5% (F)
					Risk Factor (biochemical and/or metabolic syndrome)	Elevated TC (>6.2 mmol/L)	Overall: 17% (T), 16% (M), 19% (F) Southern: 24% (T), 20% (M), 26% (F) Highlands: 16% (T), 17% (M), 15% (F) Momase: 12% (T), 10% (M), 13% (F)
						Low HDL (<1 mmol/L (M) and ≤1.3 mmol/L (F))	Overall: 55% (T), 44% (M), 64% (F) Southern: 45% (T), 31% (M), 55% (F) Highlands: 58% (T), 45% (M), 71% (F) Momase: 62% (T), 57% (M), 67% (F)
						HbA1c (≥5.7–6.4%, pre-diabetes)	Overall: 15% (T), 13% (M), 17% (F) Southern: 26% (T), 19% (M), 31% (F) Highlands: 11% (T), 12% (M), 11% (F) Momase: 7% (T), 7% (M), 7% (F)
						HbA1c (≥6.5%, T2DM)	Overall: 3% (T), 2% (M), 4% (F) Southern: 9% (T), 7% (M), 10% (F) Highlands: 0% (T), 0% (M), 0% (F) Momase: 1% (T), 0% (M), 2% (F)
					Risk Factor (physiological)	Prehypertension (SBP > 120–139.9 mmHg and/or DBP 80–89 mmHg)	Overall: 46% (T), 55% (M), 38% (F) Southern: 46% (T), 58% (M), 36% (F) Highlands: 55% (T), 58% (M), 52% (F) Momase: 38% (T), 49% (M), 29% (F)
						Hypertension (≥140/90 mmHg)	Overall: 16% (T), 19% (M), 13% (F) Southern: 22% (T), 29% (M), 17% (F) Highlands: 22% (T), 24% (M), 19% (F) Momase: 5% (T), 4% (M), 5% (F)
					NCD (self-reported diagnosis)	Cancer	Overall: 0.3% (T) Southern: 0.4% (T) Highlands: 0% (T) Momase: 0.4% (T)
						Diabetes (T2DM)	Overall: 1% (T) Southern: 2% (T) Highlands: 0% (T) Momase: 0% (T)
						CVD (stroke)	Overall: 0.4% (T) Southern: 1% (T) Highlands: 0% (T) Momase: 0% (T)
						CVD (heart disease)	Overall: 1% (T) Southern: 2% (T) Highlands: 0% (T) Momase: 0% (T)
						Chronic lung diseases (including asthma)	Overall: 2% (T) Southern: 4% (T) Highlands: 2% (T) Momase: 1% (T)
Rarau, 2019 [21]	PNG Integrated Health and Demography Surveillance System	Southern (Central Province—West Hiri), Highlands (Eastern Highlands Province—Asaro), Momase (Madang Province—Karkar Island)	Age: 15–65, *n* = 671 (not pregnant for females)	2013–2014	Risk Factor (anthropometric)	Waist circumference (≥94 cm (M) and ≥80 cm (F))	Overall: 9% (M), 52% (F) Southern: 44% (T) Highlands: 31% (T) Momase: 20% (T)
						WHR (≥0.90 (M) and ≥0.85 (F))	Overall: 61% (M), 72% (F) Southern: 51% (T) Highlands: 89% (T) Momase: 63% (T)
					Risk Factor (lifestyle)	Smoking (daily)	Overall: 65% (M), 22% (F) Southern: 36% (T) Highlands: 40% (T) Momase: 50% (T)
						Betel nut (any amount in last 30 days)	Overall: 76% (M), 80% (F) Southern: 93% (T) Highlands: 56% (T) Momase: 86% (T)
						Alcohol (any amount in the last 30 days)	Overall: 42% (M), 8% (F) Southern: 45% (T) Highlands: 22% (T) Momase: 7% (T)
						Food consumption Fruit/vegetables (consumption < 5 days/week)	Overall: 23% (M), 25% (F) Southern: 59% (T) Highlands: 7% (T) Momase: 1% (T)
						High sugar intake (>6 teaspoons of sugar daily or drinking ≥ 3 soft drinks/week)	Overall: 32% (M), 21% (F) Southern: 41% (T) Highlands: 24% (T) Momase: 9% (T)
						Salt intake (adding salt/Maggie stock cubes directly to food daily)	Overall: 70% (M), 68% (F) Southern: 86% (T) Highlands: 89% (T) Momase: 24% (T)
						Fried food (purchased or cooked at home) ≥ 5 days/week)	Overall: 67% (M), 66% (F) Southern: 92% (T) Highlands: 97% (T) Momase: 2% (T)
					Risk Factor (biochemical and/or metabolic syndrome)	Elevated TC (>6.2 mmol/L)	Overall: 16% (M), 19% (F) Southern: 23% (T) Highlands: 15% (T) Momase: 11% (T)
						Low HDL (<1 mmol/L (M) and <1.3 mmol/L (F)	Overall: 42% (M), 65% (F) Southern: 44% (T) Highlands: 61% (T) Momase: 63% (T)
						Elevated TG (≥2.3 mmol/L)	Overall: 54% (M), 56% (F) Southern: 62% (T) Highlands: 58% (T) Momase: 43% (T)
						Elevated HbA1c (≥6.5%)	Overall: 3% (M), 5% (F) Southern: 9% (T) Highlands: 0% (T) Momase: 1% (T)
						Metabolic syndrome (large WC ≥ 94 cm (M) and ≥80 cm (F) as a marker of central obesity plus any two of the following: hypertension (≥130/85 mmHg), HbA1c levels (≥6.5%), elevated TG (≥1.7 mmol/L), and low HDL-c (<1.0/mmol/L (M) and <1.3 mmol/L (F))	Overall: 6% (M), 30% (F) Southern: 27% (T) Highlands: 18% (T) Momase: 10% (T)
					Risk Factor (physiological)	Hypertension (average of the three systolic and diastolic BP of ≥130/85 mmHg and/or those on treatment for hypertension)	Overall: 38% (M), 28% (F) Southern: 40% (T) Highlands: 41% (T) Momase: 16% (T)
Peprah, 2022 [22]	PNG Demographic and Health Survey	PNG	Breastfeeding Adults Age: 15–49, *n* = 3822	2016–2018	Risk Factor (lifestyle)	Smoking (last 24 h)	Overall: 22% Southern: 21% (T) Highlands: 26% (T) Momase: 33% (T) New Guinea Islands: 20% (T)
Theilmann, 2022 [23]	PNG Demographic and Health Survey	PNG	Age: ≥15 *n* = 21,970	2016–2018	Risk Factor, (lifestyle)	Smokeless tobacco	Overall: 65% (T)
						Betel nut chewing (daily)	Overall: 65% (T)
Screeramareddy, 2022 [24]	Global Youth Tobacco Survey	PNG	Age: <18 *n* = 2301 Nationally representative schools-based survey	2016	Risk Factor (lifestyle)	Smoking (cigarette smoking in last 30 days)	Overall: 25% (T)
Choi, 2023 [25]	Global Burden of Disease	PNG	-	2019	NCD	Cancer (liver)	Prevalence: rate 95% uncertainty interval 1.4 (1.1, 1.9) (T) Incidence: rate 95% uncertainty interval 1.5 (1.2, 2) (T)
Venketasubramanian, 2021 [26]	Global Burden of Disease	PNG	Age: >18	2008–2016	NCD	CVD (stroke)	Incidence per 100,000 person-years: 159 Prevalence per 100,000: 391 (2010)
Venketasubramanian, 2021 [26]	WHO Global Health Observatory data repository	PNG			Risk Factor (anthropometric)	Obesity (BMI ≥ 30 kg/m^2^)	17% (M), 26% (F) (2016)
					Risk Factor (lifestyle)	Insufficient physical activity (<150 min of moderate intensity per week or <75 min of vigorous intensity)	11% (M), 18% (F) (2016)
					Risk Factor (biochemical and/or metabolic syndrome)	Elevated TC (TC ≥ 6.2 mmol/L)	8% (M), 10% (F) (2008)
					Risk Factor (physiological)	Hypertension (>140/90 mm Hg)	25% (M), 26% (F) (2015)
					NCD	Diabetes (T2DM) (FBG > 7 mmol/L or on medication)	15% (M), 14% (F) (2014)
Ampofo, 2020 [27]	WHO Global Health Observatory data repository	PNG	Age: >18	2014	NCD	Diabetes (T2DM)	15% (T)
Williams, 2020 [28]	Biomarkers Reflecting Inflammation and Nutritional Determinants of Anemia project	PNG	Age: 15–49 Female *n* = 738 79% rural	2005	Risk Factor (biochemical and/or metabolic syndrome)	Anaemia (hemoglobin adjusted for smoking and altitude < 12.0 g/dL).	36% (T)
						Iron deficiency (inflammation-adjusted soluble transferrin receptor > 8.3 mg/L)	8% (T)
						Vitamin A deficiency (retinol binding protein or retinol < 0.70 μmol/L)	1% (T)
Are, 2017 [29]	GLOBOCAN	PNG	-	2012	NCD	Cancer (liver)	Incidence: (age standard rate) 11 (T)
Shield, 2017 [30]	GLOBOCAN	PNG	-	2012	NCD	Cancer (lip)	Incidence: (age standard rate) 9 (T)
						Cancer (oral cavity)	Incidence: (age standard rate) 11 (T)
						Cancer (parotid and salivary gland)	Incidence: (age standard rate) 3 (T)
Capitanio, 2019 [31]	GLOBOCAN	PNG	-	2012	NCD	Cancer (kidney)	Incidence: (age standard rate) 1 (T)
Chaturvedi, 2018 [32]	GLOBOCAN	PNG	-	2018	NCD	Cancer (oral cavity)	Incidence: (age standard rate) 30 (M), 21 (F)
Goodarzi, 2020 [33]	GLOBOCAN	PNG	-	2018	NCD	Cancer (pancreatic)	Incidence (age standard rate) 2 (T)
Gunjal, 2020 [34]	GLOBOCAN	PNG	-	2018	NCD	Cancer (lip and oral cavity)	Incidence: (age standard rate) 20 (T), 28 (M), 15 (F)
Miranda-Filho, 2020 [35]	GLOBOCAN	PNG	-	2018	NCD	Cancer (lip and oral cavity)	Incidence: (age standard rate) 28 (M), 15 (F)
Mohammadian, 2020 [36]	GLOBOCAN	PNG	-	2018	NCD	Cancer (liver)	Incidence: (age standard rate) 12 (T), 14 (M), 10 (F)
Ranganath, 2021 [37]	GLOBOCAN	PNG	-	-	NCD	Cancer (pancreatic)	Prevalence: 1 (proportions per 100,000) (T) Incidence: (age standard rate) 2 (T)
Akbari, 2022 [38]	GLOBOCAN	PNG	-	2018	NCD	Cancer (gastric)	Incidence: (age standard rate) 9 (T), 12 (F), 7 (M)

‡ Unless specified; Age in years unless stated; BF = body fat; BG = blood glucose; BMI = body mass index; DBP = diastolic blood pressure; F = female; FBG = fasting blood glucose; Hb = haemoglobin; M = male; min = minute; PNG = Papua New Guinea; SBP = systolic blood pressure; T = total; TG = triglycerides; TC = total cholesterol; T2DM = type 2 diabetes mellitus; WHR = waist-to-hip ratio.

## Data Availability

Not applicable.

## Supplementary Materials

The following supporting information can be downloaded at: https://www.mdpi.com/article/10.3390/ijerph22010102/s1, Table S1: Medline Search Strategy.
