# Supplementary material for: Regional Variations in the Prevalence of Risk Factors and Non-Communicable Diseases in Papua New Guinea: A Scoping Review
Source: Int J Environ Res Public Health. 2025 Jan 14;22(1):102. doi: 10.3390/ijerph22010102 (PMC11765442; doi:10.3390/ijerph22010102)
Supplement: Supplementary file 1 [file ijerph-22-00102-s001.zip › ijerph-3400411-supplementary.pdf]

**Supplementary Table S1.** Medline Search Strategy.

| Search | Query |
|---|---|
| 1 | Papua New Guinea/ OR "Native Hawaiian or Other Pacific Islander"/ |
| | (Papua* OR New Guinea* OR PNG OR Pacific Island* OR Pacific region* OR Melanesia* OR Port Moresby OR National Capital District).tw. |
| 2 | Prevalence/ OR Incidence/ OR (Prevalence OR Incidence).tw. OR exp Risk Factors/ OR (Risk factor* OR Root cause*).tw. |
| 3 | Life Style/ OR Sedentary Behavior/ OR (Lifestyle* OR Life Style*).tw. OR (Diet or Nutrition* adj2 (Poor OR Unhealthy OR Changes OR Status OR Transition)).tw. OR (Sedentary OR Physical* inactive* OR Sitting OR Stress).tw. OR (Wellbeing adj2 (Social OR Emotional)).tw. |
| | OR exp Obesity/ OR exp Overnutrition/ OR Body Mass Index/ OR (Overweight OR Obesity OR Obese OR Obesogenic OR Excess Weight OR Adiposity OR Body Mass Index OR BMI OR Waist-hip ratio OR Waist Circumference OR Overnutrition OR Over nutrition).tw. |
| | OR Starvation/ OR Growth Disorders/ OR Malnutrition/ OR Protein-Energy Malnutrition/ OR (Starvation OR Stunting OR Stunted growth OR Undernutrition OR under nutrition OR Malnutrition OR Wasted OR Wasting OR Underweight).tw. |
| | OR Smoking/ OR (Smok* OR Tobacco).tw. |
| | OR exp Alcohol Drinking/ OR Alcohol.tw. |
| | OR Hypertension/ OR Blood Pressure/ OR Metabolic syndrome/ OR Hypertensi*.tw. OR (Blood adj2 (Pressure OR Systolic OR Diastolic OR Mean OR Arterial)).tw. OR (SBP OR DBP OR MABP).tw. |
| | OR exp Cholesterol/ OR Dyslipidemias/ OR Hyperlipidemias/ OR Hypercholesterolemia/ OR (Cholesterol OR Lipid* OR Triglyceride* OR HDL OR LDL).tw. |
| | OR Prediabetic State/ OR Glycated Hemoglobin A/ OR (Prediabet* OR BGL OR Glycated haemoglobin OR HbA1c).tw. OR (Glucose adj (Blood OR Plasma OR Intolerance OR Fasting)).tw. OR (Insulin adj (Resistance OR Fasting)).tw. |
| 4 | Noncommunicable Diseases/ OR Chronic Disease/ OR (Noncommunicable Disease* OR NCD* OR Chronic disease*).tw. |
| | OR Cardiovascular Diseases/ OR exp Heart Diseases/ OR Vascular Diseases/ OR (Cardiovascular Disease* OR CVD OR (Heart adj (Coronary OR Ischaemic OR Failure)) OR CHD OR IHD OR Coronary Artery Disease OR Atherosclerosis OR Stroke OR Haemmorrhage OR Hemorrhage).tw. |
| | OR exp Neoplasms/ OR (Cancer* OR Tumor OR Tumour OR Carcinoma* OR Metastasis OR Neoplasm OR Adenocarcinoma).tw. |

| Search | Query |
|---|---|
| | OR Pulmonary Disease, Chronic Obstructive/ OR Asthma/ OR (Respiratory disease* OR Asthma* OR Lung disease*).tw. |
| | OR Diabetes Mellitus/ OR Diabetes Mellitus, Type 2/ OR (Diabetes Mellitus OR T2D OR Type 2 diabetes OR Diabetic).tw. |
| 5 | 1 AND 2 AND (3 OR 4) |
